# Development of Communication and Language Skills in Children with Hematological–Oncological Disorders: Challenges and Perspectives

**DOI:** 10.3390/children12050574

**Published:** 2025-04-29

**Authors:** Giusy Melcarne, Giulia Marangon, Roberta Maria Incardona, Anna Agostinelli, Silvia Montino, Silvia Sorbara, Alessandra Biffi, Marta Tremolada

**Affiliations:** 1 Department of Neuroscience DNS, University of Padua, 35100 Padova, Italy; anna.agostinelli@unipd.it (A.A.); silvia.montino@unipd.it (S.M.); 2 AIL Padova ODV, 35124 Padova, Italy; robertamaria.incardona@phd.unipd.it; 3 Pediatric Hematology, Oncology and Stem Cell Transplant Center, Department of Woman’s and Child’s Health, University of Padua, 35127 Padova, Italy; giulia.marangon.10@gmail.com (G.M.); silvia.sorbara@unipd.it (S.S.); alessandra.biffi@unipd.it (A.B.); marta.tremolada@unipd.it (M.T.); 4 ANVOLT ODV, Associazione Nazionale Volontari Lotta Contro i Tumori, 20158 Milano, Italy; 5 Department of Development and Social Psychology, University of Padua, 35131 Padova, Italy

**Keywords:** language, development, communication, cancer, infants, toddlers

## Abstract

Children with onco-hematological diseases require intensive medical treatments that can affect various aspects of their development. In addition to the disease itself, what influences the course of development most are the neurotoxic effects of therapies and frequent hospitalizations, especially if they occur in the first three years of the child’s life. Among these challenges there is the potential for language delay, a condition that can impact their communication abilities and overall development. **Background/Objectives**: The aim of this study is to examine communicative and linguistic development in a small group of young children diagnosed with different forms of leukemia, rhabdomyosarcoma, and CNS tumors, recruited through the Hematology–Oncology Clinic of the Department of Child and Woman Health (University of Padova). **Methods**: Child direct (Griffiths III, PinG, PCGO) and parent indirect assessments (PVB, ABAS-II, ASCB) were provided. **Results**: Griffiths communication subscale scores in children were mainly below average (55.6%), and 44.4% attested at the clinical level in ABAS-II, with the ability to understand being significantly higher than the production of words. However, the two levels of assertiveness–responsiveness obtained balance in 66.7% of cases, and using the Griffiths personal subscale, only 22.2% of children attested below average. **Conclusions**: Understanding and addressing children’s communication needs is crucial to improve the quality of life of these young patients and foster optimal communicative and linguistic development despite the obstacles they face in order to implement interventions designed specifically for this type of population and their respective families, if necessary.

## 1. Introduction

Onco-hematological diseases, including cancers and disorders of the blood and lymphatic systems, can require rigorous and prolonged treatments. The two most common cancers in children are acute lymphoblastic leukemia (ALL) and brain tumors (BTs), with current 5-year survival rates of 84% and 75%, respectively [1]. Over time, it has been observed that cured patients can frequently report physical, cognitive, emotional, and psychological sequelae, and this has led to increased focus on the quality of life of young patients during and after their treatment [2]. These treatments, along with the stress and anxiety associated with chronic diseases, can contribute to delays in speech and language acquisition.

Speech and language skills, which develop rapidly during the first three years of life, progress at individual rates but generally follow age-related developmental milestones. However, infants and toddlers who have been treated for cancer often experience delays in reaching these milestones compared to their healthy peers, as reported by the National Institutes of Health. Children diagnosed with cancer and nonmalignant hematological disorders (NMHDs) may exhibit a range of communication disorders—including deficits in receptive and expressive language, speech, pragmatics/social language, voice, resonance, and fluency (stuttering)—that warrant intervention from speech–language pathology (SLP) professionals [3]. These impairments may be pre-existing, related to prolonged hospitalization, or directly associated with the disease or its treatments and side effects. Despite the evident presence of communication difficulties in this population and the recognized need for SLP involvement during and after treatment [4], there is currently a lack of published research specifically addressing the provision of SLP services in pediatric oncology settings [3].

### 1.1. Cognitive Sequelae Related to the Illness and Its Treatment

The cognitive functioning of children treated with anti-tumor drugs has been extensively studied by examining the long-term effects on survivors [5], as neurocognitive deficits may not become apparent immediately after treatment. Research indicates that survivors of childhood ALL may experience impairments in verbal competence, key executive functions, complex visual–spatial tasks [6], and working memory tasks [7], and a decline in both intelligence and academic achievement [8]. Survivors of both BT and ALL have shown lower-than-expected performance across various executive function tasks [1]. The main risk factors in possible developmental deficits in childhood cancer survivors include being diagnosed before the age of five [9], receiving intensity therapy, and the time elapsed since initial treatment [10]. Cognitive difficulties, such as reduced working memory and nonverbal skills, can appear within the first year of treatment, influenced by factors such as methotrexate dosage and/or infusion rate [11]. Additionally, attentional deficits are commonly observed in survivors of childhood ALL, particularly in those who underwent high-intensity treatments [12]. Hematopoietic stem cell transplantation (HSCT) can further impact cognitive outcomes, with early declines in memory and motor skills and persistent challenges in verbal and academic abilities up to three years after transplantation [13,14]. Furthermore, children treated with chemotherapy (CT) or combined chemotherapy–radiotherapy (CTRT) also tend to show lower average IQ scores compared to peers [15].

### 1.2. Language Sequelae Related to the Illness and Its Treatment

Research on the development of language and communication in pediatric cancer patients is limited and varies significantly in terms of methodology, sample characteristics, and assessment tools. However, evidence suggests that ALL survivors treated exclusively with chemotherapy may experience long-term deficiencies in language and memory functions, as well as changes in brain structure [16]. CT—especially when directed at the CNS—can have lasting effects on language development, particularly during adolescence, when linguistic demands become more complex. The most affected areas are higher-order language skills, such as understanding figurative language, resolving ambiguities, and using novel expressions, which require advanced cognitive processing [17]. Some patients also present clinically significant challenges in understanding non-literal and metaphorical language. Although basic language abilities often remain stable, the delayed emergence of difficulties in higher-level language suggests potential long-term effects that may become apparent between 5 and 20 years after treatment [17,18]. These deficits may significantly affect children’s daily functioning, particularly in educational and social contexts [18,19]. Neurocognitive impairments commonly observed in children with ALL—especially in working memory, attention, and short-term verbal memory—can contribute to language difficulties, as they hinder the ability to retain, process, and manipulate verbal information [20,21]. Preschool-aged children appear especially vulnerable, showing greater declines in working memory than those diagnosed at older ages [22] and scoring significantly lower than their healthy peers in areas such as socialization, communication, and both language comprehension and expression [23]. Although deficits in figurative language have been observed in children following CNS-directed CT, some studies have not found significant differences in descriptive case analysis or error patterns [24]. As higher-order language skills continue to develop beyond early childhood, ongoing monitoring of language development in ALL survivors is crucial. Intrathecal chemotherapy (ITC) has been associated with language impairments [25,26,27], although findings are not consistent across all studies [17,28]. Late effects of cancer treatment may persist for decades, with some research indicating deterioration in language abilities even 30 years after treatment [29,30]. Despite this, much of the existing literature has focused on outcomes within the first five years after treatment [25,26,27], although emerging data suggest that neurocognitive and language-related challenges may also appear or worsen beyond this period [2].

### 1.3. Hospitalization and the Risk of Communicative Linguistic Deprivation

The effect of illness, hospitalization, and medical treatments on a child’s development and adaptive functioning vary widely depending on factors such as the nature of the illness, the child’s age, cognitive and social abilities, family resources, and availability of medical, family, and social support. Both short- and long-term consequences may arise as a result of physical, communicative–linguistic, and psychological distress associated with the disease and prolonged hospital stays [23]. Short-term effects include fatigue, nausea, ulcers, taste changes, appetite loss, infections, anemia, bleeding, and hair loss [5]. In the long term, sequelae may include both physical and psychosocial disorders, influenced by the type of illness, intensity of treatment, the child’s age, and lack of social interaction [5,8,9,10]. These outcomes can affect physical, cognitive, and social development, influencing motor coordination, attention, communication, academic achievement, and peer interaction, with potential long-lasting effects on overall future quality of life [1,25,26]. Hospitalization may also lead to linguistic deprivation, particularly in children with restricted communication due to medical conditions, sedative use, or cognitive impairments. Reduced verbal interaction with caregivers and healthcare professionals can hinder both cognitive and language development. Cancer treatment may further delay developmental milestones, with children diagnosed before the age of four showing slower progression in vocabulary acquisition, cognitive skills, and motor development compared to healthy peers [31,32]. Furthermore, the disruption of family dynamics during hospitalization often leads to parental feelings of guilt and insecurity, which may result in altered perceptions of the child’s autonomy and overprotective parenting behaviors [33]. Pediatric cancer survivors are at continued risk of reduced health-related quality of life (HRQoL) due to persistent language-related disabilities and limited access to supportive social networks [34].

### 1.4. Research Aims and Questions

This pilot study aims to assess the language and personal development of infants and toddlers who have undergone treatment for onco-hematological diseases, adopting a multi-method and multi-informative approach.

Based on the known effects of illness and medical interventions, we hypothesize that participants may exhibit lower performance in language-related tasks. The study seeks to identify whether potential delays are more pronounced in receptive or expressive language domains and to evaluate the balance between assertiveness and responsiveness in communication. Additionally, the study explores the socio-personal domain to determine whether the experience of illness may have contributed to developmental regression or increased dependence on caregivers.

## 2. Materials and Methods

### 2.1. Procedures

The study design was approved by Padua Hospital Ethical Committee on 16 September 2024 (code: 6054/AO/24).

Children and their parents were invited to participate in the study by the clinic’s clinical psychologist, who had an existing therapeutic relationship with the families through prior counseling and support sessions. Following the provision of informed consent, the psychologist introduced the speech and language therapist during the stay of the families in the hospital, either in the day hospital or inpatient unit. Assessment sessions were scheduled in agreement with the parents. All participants’ parents were proficient in the Italian language.

### 2.2. Participants

The study was carried out at the pediatric department of the Hematology–Oncology Clinic of the Department of Child and Woman Health (University of Padua) in the year 2024 within the Stai Bene 3.0 Plus! project, subsidized by AIL Padova ODV and the Italian Ministry of Labor and Social Policies.

The inclusion criteria were as follows:

- Diagnosis of BT or LL.
- Age range between 0 and 32 months.
- Patient at the pediatric department of the Hematology–Oncology Clinic of Padua’s Paediatric Hospital.
- Italian speaker and monolingual.

Patients with diagnoses of neurodevelopmental disorders were excluded. In this study, 9 children (6 males and 3 females) participated, with an average age of 21.22 months (SD = 8.46). Participants were treated for rhabdomyosarcoma (n. 2), germinoma (n. 1), low-grade glioma (n. 1), and acute lymphoblastic (n. 4) and myeloid leukemias (n. 1). Two of them have siblings.

### 2.3. Instruments

Firstly, data were collected through an indirect assessment by administration of three standardized tools to parents: Adaptive Behavior Assessment System Second Edition (ABAS-II)—communication and socialization subscale, the Child’s Socio-Conversational Skills (ASCB), and the Child’s First Vocabulary McArthur (PVB). Then, a direct assessment was conducted by an expert psychologist using the Griffiths subscale and by an expert speech and language therapist through “Parole in Gioco (PinG)” and “Prova di Comprensione Grammaticale con Oggetti (PCGO)”. These tests are designed with age-, gender-, and language-appropriate norms, allowing for meaningful comparison between the patients’ performance and that of typically developing children in the general population. This approach provides a valid benchmark for identifying potential developmental or functional delays.

#### 2.3.1. Griffiths—Language and Communication Subscale

The Griffiths III provides a comprehensive assessment of a child’s overall developmental profile. The scales are designed to measure developmental trends that are indicative of intellectual functioning and mental growth in infants and young children. The language and communication subscale (B) evaluates overall language development, encompassing expressive and receptive language abilities, as well as, to a lesser extent, the social use of language. This subscale (B) has been refined to more clearly differentiate between expressive and receptive language skills, as well as syntactic, semantic, and pragmatic components, allowing for more nuanced interpretation. Additionally, memory has been integrated into this subscale. The personal–social–emotional subscale (D) assesses key aspects of the child’s emerging sense of self and autonomy, social interactions, and emotional development.

The Italian adaptation of the Griffiths III has demonstrated excellent psychometric properties. Internal consistency was high across all scales, with Cronbach’s alpha values ranging from 0.83 to 0.99 depending on the scale and age group. Test–retest reliability was similarly strong, showing stability over time with coefficients between 0.96 and 0.99 across the various scales. Furthermore, the Italian version exhibited robust constructs and convergent and discriminant validity, confirming its effectiveness in measuring intended constructs while clearly distinguishing between related and unrelated domains [35]. Age- and gender-specific normative data allow for precise calculation of weighted scores, enabling direct comparison between the child being assessed and same-age, same-gender peers from the normative sample.

#### 2.3.2. Adaptive Behavior Assessment System Second Edition (ABAS-II) Parent Form (Ages 0–5)—Communication and Socialization Subscale

The Adaptive Behavior Assessment System Second Edition (ABAS-II) gives a complete picture of adaptive skills throughout the lifespan. The ABAS-II covers four broad adaptive domains: conceptual (DAC), social (DAS), practical (DAP), and general adaptive composite (GAC) (mean = 100; SD = 15). The GAC enables a comparison of the evaluated person’s adaptive skills with those of other individuals in the same age group from the normative sample. Within these domains, it assesses 10 adaptive skill areas (communication, community use, functional pre-academics, home/school living, health and safety, leisure, self-control, socialization, and motor) (mean = 10; SD = 3). On a four-point response scale, raters indicate whether the individual can perform each activity and, if so, how frequently they perform it when needed. The communication subscale investigates listening, comprehension, and production skills necessary for communication with other people. The socialization scale analyzes the skills needed to socialize and get along with people. The ABAS-II is a reliable instrument with excellent psychometric properties. Its internal consistency is high, with reliability coefficients ranging from 0.97 to 0.99 for the GAC, from 0.91 to 0.98 for the three adaptive behavior domains, and from 0.92 to 0.99 for the skill areas. Test–retest reliability coefficients range from 0.80 to 0.90 for the GAC and the three domains; the test–retest reliability coefficients range from 0.70 to 0.90 for the skill areas. The construct validity of the test is very strong [36].

#### 2.3.3. The Child’s Socio-Conversational Skills (ASCB)

The Child’s Socio-Conversational Skills questionnaire is a clinical instrument that involves parents in the indirect assessment of children aged 12 to 36 months with communication and language disorders. It has a solid foundation in the social-interactionist theoretical approach towards the exchange of information between interlocutors, which requires a certain degree of reciprocity and bidirectionality with active involvement of partners. It consists of two scales—the assertiveness scale and the responsiveness scale—with a total of 25 questions. The assessment of the reliability of the scales on the sample of Italian children was conducted by calculating the Cronbach alpha coefficient separately for the items of responsiveness and for the items of assertiveness. The results of this analysis show an alpha coefficient value of 0.93 for the 15 items of assertiveness and 0.92 for the 10 items of responsiveness [37].

#### 2.3.4. The Child’s First Vocabulary McArthur (PVB)

The purpose of the Child’s First Vocabulary questionnaire is to gather information on the course of the child’s communicative and linguistic development, starting from the first nonverbal signals through to the expansion of vocabulary, the emergence of grammar, and the first word combinations. Given the strong changes that occur between the first and third years of age, it was necessary to formulate two worksheets: “Gestures and Words”, for children between 8 and 17 months, and “Words and Sentences”, for those between 18 and 30 months. Parents are instructed to complete the questionnaire, which takes from 20 to 40 min, depending on the child’s communication level and age [38].

#### 2.3.5. Parole in Gioco (PinG)

PinG is a direct observation tool for specific language, targeting children with communicative and language development levels between approximately 19 and 37 months of age. It allows for the identification and assessment of children with a specific language delay or disorder and/or the description of the language profile of children with language disorders associated with sensory or cognitive deficits. The material consists of 2 sets of color photographs: one assesses children’s ability to comprehend and produce nouns (noun comprehension subtest and noun production subtest) and one the ability to comprehend and produce verbs (verb comprehension subtest and verb production subtest). The material is well suited for use with young children, as the manipulable photographic stimuli are engaging and help elicit interest. These images also aid in the production of words whose underlying lexical representations may not yet be fully stabilized. In the PinG assessment, each subtest is scored by summing the number of correct responses. Internal consistency was evaluated using Cronbach’s alpha, which indicated excellent reliability (≥0.80) for three of the four subtests, while the verb comprehension subtest demonstrated lower, though still acceptable, reliability. Test–retest reliability was examined trough split-half analysis, in which each subtest’s items were divided into two equal halves. The analysis revealed good repeatability across all subtests, with stronger performance observed in the production subtests (correlation close to 0.80) compared to the comprehension (correlation of 0.68 for NC and 0.50 for VC). Additional Cronbach’s alpha estimates reconstructed from the split halves were also satisfactory, although the VC subtest consistently yielded slightly lower values compared to the other three. To further assess test stability and internal consistency, Pearson’s r coefficient was calculated to examine correlations between the scores of the four subtests across the sample. All correlations were statistically significant, with particularly strong associations shown between the two production subtests (r = 0.661). Overall, the production subtests demonstrated the highest correlations with the remaining subtests [39].

#### 2.3.6. Prova di Comprensione Grammaticale con Oggetti (PCGO)

The PCGO is a direct observational tool designed to reliably assess grammatical comprehension in children aged 18 to 48 months. It is particularly suited for clinical settings and evaluates the child’s ability to understand simple sentences through the use of familiar three-dimensional objects. The assessment involves presenting the child with sentences of increasing grammatical complexity, based on contemporary developmental models, and prompting responses in the form of object manipulation. This method—requiring the child to act upon specific sets of objects—has been deemed especially appropriate for young children and those with developmental disorders. During the procedure, the clinician delivers stimulus sentences of gradually increasing difficulty, encouraging the child to either identify the named object or enact the sentence using the available objects. Scoring is based on the number of correct responses across three categories: total score, nuclear phrases, and grammatical phrases. Internal consistency was evaluated using Cronbach’s alpha coefficient, with results indicating excellent reliability across all scoring categories (total score: α = 0.91; nuclear phrases: α = 0.88; grammatical phrases: α = 0.90) [40].

## 3. Results

**Figure 1:**
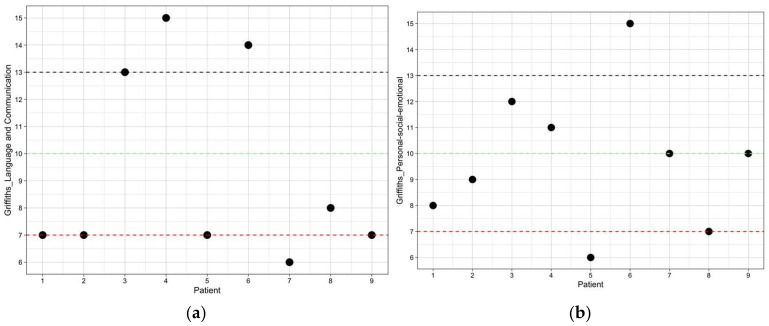
(**a**) Distribution of language PS. (**b**) Distribution of personal–social–emotional PS.

After the inclusion criteria were applied, the final study sample consisted of nine participants. The mean value of age in months at diagnosis was 17 (SD 6.44, 8–25), while the mean value of age in months at Griffiths assessment was 21.56 (SD 6.31, 13–31). The average score obtained in Griffiths language subscale was 9.33 (SD 3.57, 6–15), while the average score found in Griffiths language subscale score (PS) was 40.89 (SD 36.730, 12–93). More specifically, the performances of five of the children (55.6%) were below average, one (11.1%) average, two (22.2%) above average, and one (11.1%) high. The average value of the Griffiths language subscale development quotient was 96.89 (SD 17.29, 82–123) (Figure 1a). The mean value of the Griffiths personal–social–emotional subscale obtained was 9.78 (SD 2.73, 6–15), while the mean value of the Griffiths personal–social–emotional subscale score (PS) was 45.44 (SD 28.65, 9–94). More specifically, the performances of two of the children (22.2%) were below average, six (66.7%) average, and one (11.1%) above average. The average value of the Griffiths personal–social–emotional subscale development quotient was 98.67 (SD 13.59, 80–123) (Figure 1b).

Regarding the ASCB questionnaire, four children (44.4%) showed an absent assertiveness, four children (44.4%) an emerging assertiveness, and only one (11.1%) a developed assertiveness. Then, while three children (33.3%) showed an absent level of responsiveness, five children (55.6%) showed emerging responsiveness and only one (11.1%) showed a developed responsiveness. The two levels appear balanced in 66.7% of cases (n.6), while they do not appear balanced in 33.3% of cases (n.3).

Regarding the understanding of words registered with the PVB questionnaire, one child (11.1%) was in the range between the 10th and 25th percentile, three cases (33.3%) were in the 25th percentile, one (11.1%) was in the 5th percentile, three cases (33.3%) were in the 50th percentile, and only one (11.1%) reached the 95th percentile. So only two cases (22.2%) ranked between 0 and the 25th percentile, while seven children (77.8%) had performance higher or equal to the 25th percentile.

**Figure 2:**
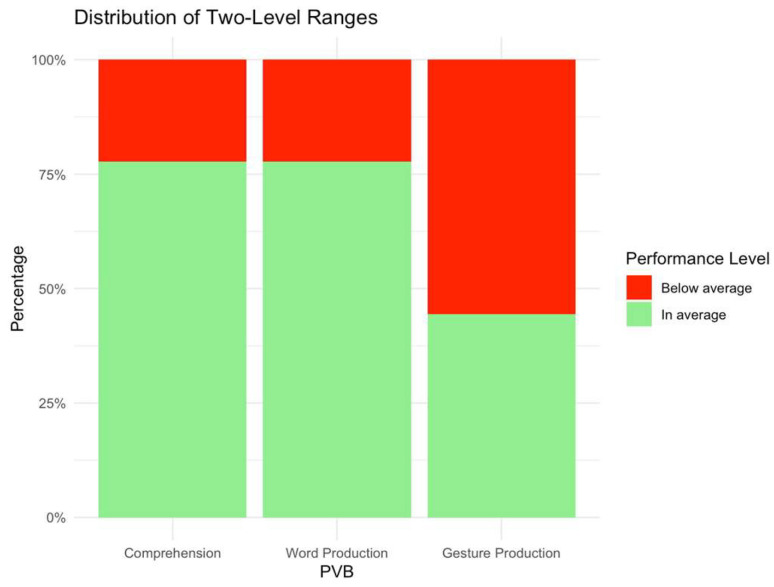
Distribution of two-level ranges in PVB scores.

Concerning the production of words registered with the PVB questionnaire, two children (22.2%) scored below the 5th percentile, one child (11.1%) reached the 10th percentile, one case (11.1%) was in the 25th percentile, two children (22.2%) were in the 5th percentile, two cases (22.2%) were in the 50th percentile, and only one (11.1%) reached the 95th percentile. Therefore, five cases (55.6%) ranked between 0 and the 25th percentile, while four (44.4%) had performance above the 25th percentile. Relative to the production of gestures registered with the PVB questionnaire, one child (11.1%) scored below the fifth percentile, one child (11.1%) reached the 10th percentile, two cases (22.2%) were in the 25th percentile, four children (44.4%) were in the 50th percentile, and only one (11.1%) reached the 95th percentile. Therefore, as with the word comprehension task, only two cases (22.2%) ranked between 0 and the 25th percentile, while seven (77.8%) had higher or equal performance to the 25th percentile (Figure 2).

**Figure 3:**
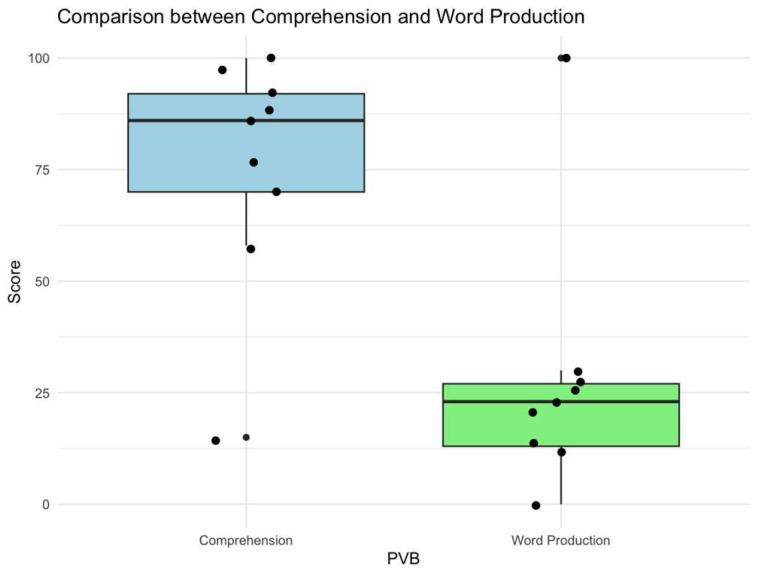
Significative comparison between comprehension and word production obtained by Wilcoxon non-parametric test.

The average word comprehension value is 75.89 (SD 26.455; 15–100), while the average word production value is 27.67 (SD 28.688, 0–100). The analysis performed by the Wilcoxon non-parametric test (Z = −2.52; *p* = 0.01) suggested that the ability to understand was significantly higher than the production of words in the analyzed sample (Figure 3).

With regard to the communication and socialization subscales of the ABAS-II, three cases (33.3%) achieved a score of 6, one case (11.1%) a score of 7, one case (11.1%) a score of 12, two cases (22.2%) a score of 9, and two cases (22.2%) a score of 10. More specifically, the performance of four children (44.4%) was below average, while the remaining five children (55.6%) showed an average performance.

A direct assessment with linguistic evaluation by the speech therapist was then conducted only for those children who scored below average on the Griffiths language subscale and/or obtained a deficient score (below the fifth percentile) for the production of words registered with the PVB questionnaire (a total of six children, 66.67%).

**Figure 4:**
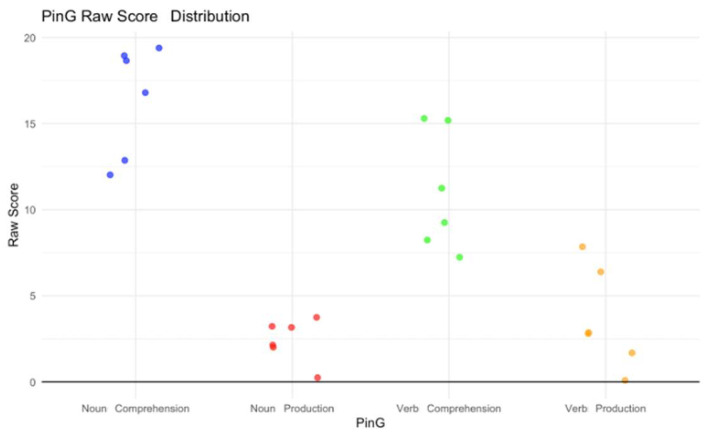
PinG raw score distribution along all the subscales.

The average score on the PCGO test that assessed verbal comprehension was 40 (SD 30.822, 0–90). The average score on the PCGO-I (nuclear phrases) was 60 (SD 25.577, 25–90). The average score on the PCGO-II (grammatical phrases) was 40 (SD 32.404, 5–90). Regarding the PinG test, the average score obtained on the noun comprehension subscale was 49.17 (SD 36.935, 5–90). The average value of range was 0.67 (SD 0.516). Only two cases (22.2%) ranked between 0 and the 25th percentile, while four (44.4%) had performance equal to or higher than the 25th percentile. Regarding the noun production subscale, five children (55.6%) showed low results (lower than the 5th percentile); in particular, one (11.1%) showed results much lower than the 5th percentile. Only one case showed better performance, although this was not in line with what was expected by chronological age (10th percentile). The average score obtained on the verb comprehension subscale was 14.17 (SD 8.612, 5–25). The average value of range was 0.33 (SD 0.516). Most of the patients showed low results; in particular, performance was measured in the 5th percentile in one case (11.1%) and the 10th percentile in three children (33.3%), while two cases (22.2%) showed better performance (25th percentile). Regarding the verb production subscale, half of the sample performed in the tenth percentile, while the others were below the fifth percentile (Figure 4).

## 4. Discussion

Speech and language skills undergo rapid development during the first three years of life, although the rate of progression may vary among individual children [41]. Typically, these abilities follow age-related developmental milestones. However, infants and toddlers who have undergone cancer treatment often exhibit delays in achieving certain milestones compared to their healthy peers [32]. In the present study, 55.6% of children performed below average on the Griffiths communication subscale and 44.4% attested at clinical level in ABAS-II. In this sample, the assertiveness–responsiveness levels investigated were found to be balanced in 66.7% of cases, although at an emergent level. These data suggest that children with oncological and hematological conditions, despite isolation and challenges, develop emerging assertiveness and responsiveness skills that allow them to appropriately express their needs and desires, such as the need for emotional support, medical treatment, or social interaction, while also responding to the needs of others in caregiving and relational contexts. Caregiver support is crucial to model this process, as children rely on parents to foster these skills [4]. By creating a safe and responsive environment, caregivers help promote an open and balanced form of communication. “The Clinical Practice Guideline for the Management of Communication and Swallowing following Childhood Brain Tumour and Leukaemia” recommends that communication assessment and intervention be provided to children diagnosed with brain tumors or leukemia [42]. This recommendation is based on the high prevalence of communication difficulties observed in this population, which may include mutism, speech production deficits, and impairments in oral language skills. Such difficulties can emerge at various stages, including at the time of diagnosis, during treatment, or even months or years after the conclusion of medical interventions [43]. A key aspect to note is the significant discrepancy between comprehension and production observed in the sample: children showed strong receptive skills, while their verbal production levels were notably deficient, consistent with what is reported in the literature [30]. In fact, most children in the study (55.6%) showed below-average scores for producing nouns and the whole sample showed difficulties with verbs. This could be due to motor or neurological difficulties related to the illness or treatments that can affect the language production process (namely the ability to articulate words correctly) without compromising comprehension. Articulation is a cognitively demanding language activity, involving several stages (formulation of meaning, selection of the right words, activation of their phonological information, and, finally, the use of the motor system to produce the sounds); children could successfully go through these stages but face difficulties in the final one, due to the considerable cognitive effort already required [34]. Additionally, psychological factors such as anxiety, fear, or a lack of stimulating social interactions during the treatment phases could also negatively affect their desire or ability to communicate verbally [23]. However, only 22.2% scored below average on the socialization scale, probably due to the fact that social skills in this age group (0–32 months) are typically very basic and foundational, gradually becoming more complex and developed over time. SLPs should be equipped with the knowledge and skills to support these children, by fostering interactions that promote speech, language, and communication, such as shared book reading (SBR) and developmentally appropriate interactive play, while actively involving parents as key partners in the intervention process [4]. Parent-led, child-centered interventions, including SBR, can significantly enhance early language development by improving the quality of parent–child interactions [44]. Such programs have been shown to boost expressive vocabulary in late talkers and promote more responsive and less directive communication styles in parents. In children with developmental language disorders, home-based SBR interventions have also led to positive changes in both parental and child communicative behaviors, highlighting the potential of involving parents directly in early language support [44,45]. Furthermore, families should receive early and ongoing education about common and long-term communication difficulties. This helps them recognize issues, seek help, and advocate for the child’s needs, both shortly after treatment and in the future [42]. Targeted support from healthcare professionals and speech–language therapists can significantly improve communication abilities in children with onco-hematological conditions, allowing them to preserve social interactions and better manage the emotional and cognitive challenges associated with their illness [46]. Such interventions not only enhance immediate clinical outcomes but also contribute positively to long-term developmental pathways [16]. Evidence shows that psychological support and parent education can help improve HRQL in this population [47]. Therefore, integrating routine psychological assessments and providing targeted psychosocial support are essential to help children and their families manage stress and foster adaptive coping strategies throughout and beyond the treatment period. Moreover, multidisciplinary, family-centered rehabilitation approaches have demonstrated promise in promoting overall well-being and facilitating reintegration into daily life [47,48].

Our study has some limitations. First, the lack of a control cohort, which would have allowed us to compare our samples’ developmental trajectories with those of healthy children in order to observe how therapies and hospitalization affect the language communication development in young children of the same age. Our methodological choice, also adopted in similar research [49,50], allows us to compare our sample to children who are developing in line with Italian norms [35,36,37,38,39,40]. Furthermore, the limited number of patients included in the study represents another important limitation. At this exploratory phase, we decided to consider all diagnoses together. Once the sample of young patients becomes larger, we will be able to better differentiate patient performance by type of treatment. However, the existing literature on this topic often includes small sample sizes [17,18,24], due to the lower incidence of disease and the challenges associated with conducting research in this population, especially in very young age groups (infants and toddlers). The vulnerability of these patients and the complexity of their medical treatments often make recruitment more challenging, as it requires careful ethical considerations and often depends on parental consent during highly stressful periods. Studies describing the linguistic functioning of larger samples have less restrictive inclusion criteria with respect to the age range [29]. Despite the reduced sample size, focusing on this specific age group provides critical insights that broader studies may overlook, helping inform more tailored and effective care strategies for this specific population.

Future prospects for research in this field require the expansion of the study sample to include a greater number of participants and a more balanced representation of socio-demographic variables. In addition, the study group planned to carry out the study to monitor the long-term effects of oncology treatments and hospitalization and to possibly follow the positive effects of language stimulation on children in this age group. Longitudinal studies that monitor the development of children in the different stages of therapeutic pathways, or even at a later stage, would be critical for observing the short- and long-term effects of therapies and hospitalization.

## 5. Conclusions

The management of communicative and linguistic development in children with onco-hematological diseases requires a multidisciplinary approach involving healthcare professionals, speech therapists, and psychologists. Some authors have pointed out that communicative–linguistic difficulties are sensitive to rehabilitation [2,31]. Individualized language intervention can promptly begin when a child’s performance deviates from its expected trajectory [30]. Early language stimulation, including parental education, adapting communication strategies to the child’s abilities, and providing psychological support to cope with the emotional stress associated with the illness and its treatments, can help mitigate potential delays and difficulties, promoting communicative and linguistic development, which is critical for positive quality-of-life outcomes.

## Data Availability

The original contributions presented in this study are included in the Supplementary Materials. Further inquiries can be directed to the corresponding author.

## Supplementary Materials

The following supporting information can be downloaded at https://www.mdpi.com/article/10.3390/children12050574/s1, Table S1: Data.
