# Supplementary material for: Development of Communication and Language Skills in Children with Hematological–Oncological Disorders: Challenges and Perspectives
Source: Children (Basel). 2025 Apr 29;12(5):574. doi: 10.3390/children12050574 (PMC12109730; doi:10.3390/children12050574)
Supplement: Supplementary file 1 [file children-12-00574-s001.zip › children-3585222-supplementary.pdf]

DATASET Development of Communication and Language Skills in children with Hematologic-Oncological Disorders: Challenges and Perspectives

GENDER	DIAGNOSIS	DIAGNOSIS_NUM	AGE_AT_DIAGN	GRIFFITHS_DATE	AGE_AT_EVALUATIO	TIME_FROM_	EQUIVALENT_AG	GRIFFITHS_B_PG	GRIFFITHS_B_PP	GRIFFITHS_B_PERC	GRIFFITHS_B_QDS	B_RANGE	B_RANGE_NUM	B_RANGE_2_LIVELLI	ITEMB1_1	ITEMB1_2	ITEMB1_3	ITEMB1_4	ITEMB1_5	ITEMB1_6	ITEMB1_7
1	RHABDOMYOSARCOMA RE	1	10	11/4/2023	13	1	9	13	7	16	86	BELOW AVERAGE	-1	0	1	1	1	1	1	1	1
1	ALL (Acute Lymphoblastic Le	0	23	2/21/2024	31	9	26	30	7	13	83	BELOW AVERAGE	-1	0	1	1	1	1	1	1	1
1	RHABDOMYOSARCOMA RE	1	17	12/5/2023	18	0	24	27	13	86	116	ABOVE AVERAGE	1	1	1	1	1	1	1	1	1
2	GERMINOMA	1	25	4/30/2024	28	3	35	43	15	93	123	HIGH	2	1	1	1	1	1	1	1	1
1	ALL (Acute Lymphoblastic Le	0	14	6/27/2024	15	1	11	15	7	19	87	BELOW AVERAGE	-1	0	1	1	1	1	1	1	1
1	ALL (Acute Lymphoblastic Le	0	19	8/13/2024	19	1	25	29	14	90	120	ABOVE AVERAGE	1	1	1	1	1	1	1	1	1
2	LOW-GRADE GLIOMA	1	8	4/24/2024	18	10	13	18	6	12	82	BELOW AVERAGE	-1	0	1	1	1	1	1	1	1
2	AML (Acute Myeloid Leukemi	0	12	5/9/2024	26	14	23	29	8	21	89	AVERAGE	0	1	1	1	1	1	1	1	1
1	ALL (Acute Lymphoblastic Le	0	25	9/17/2024	26	1	25	29	7	18	86	BELOW AVERAGE	-1	0	1	1	1	1	1	1	1

ITEMB1_8	ITEMB1_9	ITEMB1_10	ITEMB1_11	ITEMB1_12	ITEMB1_13	ITEMB1_14	ITEMB1_15	ITEMB1_16	ITEMB1_17	ITEMB2_1	ITEMB2_2	ITEMB2_3	ITEMB2_4	ITEMB2_5	ITEMB2_6	ITEMB2_7	ITEMB2_8	ITEMB2_9	ITEMB2_10	ITEMB2_11	ITEMB2_12	ITEMB2_13	ITEMB2_14	ITEMB2_15	ITEMB2_16	ITEMB3_1	ITEMB3_2	ITEMB3_3	ITEMB3_4
1	1	1	1	1	1	0	0	0	0																				
1	1	1	1	1	1	1	1	1	1	1	1	1	1	1	1	1	1	1	1	1	0	1	1	0	0	0	0		
1	1	1	1	1	1	1	1	1	1	1	1	1	1	1	1	1	1	1	0	1	0	0	0	0					
1	1	1	1	1	1	1	1	1	1	1	1	1	1	1	1	1	1	1	1	1	1	1	1	1	1	1	1	0	1
1	1	1	1	1	0	0	1	0	1	0	1	0	0	0	0														
1	1	1	1	1	1	1	1	1	1	1	1	1	1	1	1	1	1	1	1	0	1	1	0	0	0	0			
1	1	1	1	1	1	1	1	1	1	1	0	0	0	0															
1	1	1	1	1	1	1	1	1	1	1	1	1	1	1	1	1	1	1	1	1	0	1	0	0	0	0			
1	1	1	1	1	1	1	1	1	1	1	1	1	1	1	1	1	1	1	0	1	1	1	0	0	0	0			

ITEMB3_5	ITEMB3_6	ITEMB3_7	ITEMB3_8	ITEMB3_9	ITEMB4_1	ITEMB4_2	ITEMB4_3	ITEMB4_4	ITEMB4_5	ITEMB4_6	ITEMB4_7	ITEMB4_8	ITEMB4_9	EQUIVALENT_	GRIFFITHS_D_PG	GRIFFITHS_D_PP	GRIFFITHS_D_PERC	GRIFFITHS_D_QDS	D_RANGE	D_RANGE_NUM	D_RANGE_2	ITEMD1_1	ITEMD1_2	ITEMD1_3	ITEMD1_4	ITEMD1_5
														12	21	8	21	89	AVERAGE	0	1	1	1	1	1	1
														29	37	9	32	93	AVERAGE	0	1	1	1	1	1	1
														23	31	12	76	112	AVERAGE	0	1	1	1	1	1	1
0	1	1	1	1	1	1	0	1	0	0	0	0		29	40	11	61	105	AVERAGE	0	1	1	1	1	1	1
														11	20	6	9	80	BELOVE AVERAGE	-1	0	1	1	1	1	1
														26	35	15	94	123	HIGH	2	1	1	1	1	1	1
														18	29	10	53	101	AVERAGE	0	1	1	1	1	1	1
														21	32	7	16	85	BELOVE AVERAGE	-1	0	1	1	1	1	1
														26	35	10	47	100	AVERAGE	0	1	1	1	1	1	1

ITEMD1_6	ITEMD1_7	ITEMD1_8	ITEMD1_9	ITEMD1_10	ITEMD1_11	ITEMD1_12	ITEMD1_13	ITEMD1_14	ITEMD1_15	ITEMD1_16	ITEMD1_17	ITEMD1_18	ITEMD2_1	ITEMD2_2	ITEMD2_3	ITEMD2_4	ITEMD2_5	ITEMD2_6	ITEMD2_7	ITEMD2_8	ITEMD2_9	ITEMD2_10	ITEMD2_11	ITEMD2_12	ITEMD2_13	ITEMD2_14	ITEMD2_15	ITEMD3_1	ITEMD3_2
1	1	1	1	1	1	1	1	1	0	1	1	1	1	1	1	1	1	1	0	0	0	0							
1	1	1	1	1	1	1	1	1	1	1	1	1	1	1	1	1	1	1	1	1	1	1	1	1	1	1	1	1	1
1	1	1	1	1	1	1	1	1	1	1	1	1	1	1	1	1	1	1	1	0	1	0	1	1	0	1	1	1	0
1	1	1	1	1	1	1	1	1	1	1	1	1	1	1	1	1	1	1	1	1	1	1	1	1	0	1	1	0	0
1	1	1	1	1	1	1	1	1	0	1	1	1	1	0	0	1	1	0	0	0	0								
1	1	1	1	1	1	1	1	1	1	1	1	1	1	1	1	1	1	1	1	1	1	1	1	1	1	1	1	0	1
1	1	1	1	1	1	1	1	1	1	1	1	1	1	1	1	1	0	1	0	1	1	1	1	1	0	1	0	0	0
1	1	1	1	1	1	1	1	1	1	1	1	1	1	1	1	1	1	1	1	1	1	1	1	1	0	1	1	0	0
1	1	1	1	1	1	1	1	1	1	1	1	1	1	1	1	1	1	1	1	1	1	1	1	1	1	1	1	0	0

ITEMD3_3	ITEMD3_4	ITEMD3_5	ITEMD3_6	ITEMD3_7	ITEMD3_8	ITEMD3_9	ITEMD3_10	ITEMD4_1	ITEMD4_2	ITEMD4_3	ITEMD4_4	ITEMD4_5	ITEMD4_6	ITEMD4_7	ITEMD4_8	ITEMD4_9	ITEMD4_10	ASCB_DATE	ASCB_AGE	TIME_FROM_DIAG	ASSERTIVENESS_T	ASSERTIVITA_AVERAG	ASSERTIVIENESS_LEVE	ASSERTIVENESS_LEVEL_NUM
																		12/14/2023	12	3	38	2.5	ABSENT	0
1	0	1	0	0	0	0												3/25/2024	32	10	52	3.5	EMERGENT	1
0	0	0																12/5/2023	18	0	45	3	EMERGENT	1
0	1	1	1	1	0	1	0	1	0	1	1	0	0	0	0			4/29/2024	27	3	56	3.7	EMERGENT	1
																		7/28/2024	16	0	39	2.6	ABSENT	0
1	0	0	0	0														9/5/2024	20	2	53	3.5	EMERGENT	1
0																		4/12/2024	17	10	39	2.6	ABSENT	0
0	0																	4/26/2024	25	14	39	2.6	ABSENT	0
1	0	0	1	0	0	0	0											9/10/2024	26	1	61	4.1	DEVELOPED	2

RESPONSIVENESS_	RESPONSIVENESS_AVE	RESPONSIVENESS_LEVEI	RESPONSIVENESS_LEVEL_NU	BALANCE	BALANCE_NUMERIC	PVB_24_DATE	PVB_24_AGE	TIME_FROM_DIAGNOSIS	COMPREHENSION_24	COMPREHENSION_PERCEN	COMPREHENSION_RANGE_2_LEVELS	WORD_PRODUCTION_24	WORD_PRODUCTION_PERCENTAG
34	3.4	EMERGENT	1	NO	0	12/14/2023	12	3	70	95	1	27	95
34	3.4	EMERGENT	1	YES	1	3/22/2024	32	10	97	50	1	11	<5
32	3.2	EMERGENT	1	YES	1	12/5/2023	18	0	58	10-25	0	23	50
41	4.1	DEVELOPED	2	NO	0	4/9/2024	27	3	100	50	1	100	50
20	2	ABSENT	0	YES	1	7/28/2024	16	0	15	5	0	0	<5
37	3.7	EMERGENT	1	YES	1	9/5/2024	20	2	77	25	1	25	25
20	2	ABSENT	0	YES	1	4/12/2024	17	10	86	25	1	30	10
20	2	ABSENT	0	YES	1	4/26/2024	25	14	88	25	1	13	5
39	3.9	EMERGENT	1	NO	0	9/10/2024	26	1	92	50	1	20	5

WORD_PRODUCTION_RANGE_2_LEVELS	GESTURE_PRODUCTION_	GESTURE_PRODUCTION_PERC	GESTURE_PRODUCTION_RANGE_2_LEVE	ABAS_DATE	ABAS_AGE	TIME_FROM_DIAGNOSIS_	COMMUNICATION_GR	COMMUNICATION_PEF	COMMUNICATION_RANGE	COMMUNICATION_RANGE_NUME	ABAS_COMMUNICATION_RANG	PinG_DATE	PinG_AGE
1	15	95	1	2/28/2024	15	5	23	6	ABOVE AVERAGE	-1	0		
0	12	10	0	2/21/2024	31	9	49	6	ABOVE AVERAGE	-1	0	3/26/2024	32
1	14	25	1	12/5/2023	18	0	36	9	AVERAGE	0	1		
1	17	50	1	4/9/2024	27	2	64	12	AVERAGE	0	1		
0	4	<5	0	7/4/2024	15	2	21	7	ABOVE AVERAGE	-1	0	12/28/2024	21
1	17	50	1	8/13/2024	19	1	35	10	AVERAGE	0	1	9/18/2024	21
0	17	50	1	4/22/2024	18	9	36	10	AVERAGE	0	1	9/24/2024	23
0	17	50	1	5/14/2024	26	14	44	9	AVERAGE	0	1	6/5/2024	26
0	15	25	1	9/18/2024	26	1	43	6	ABOVE AVERAGE	-1	0	9/24/2024	26

TIME_FROM_DIAGNOSIS_	NAME_COMPREHENSION_G	NAME_COMPREHENSION_I	NAME_COMPREHENSION_RANGE_2_LEVE	NAME_PRODUCTION_GR	NAME_PRODUCTION_PE	NAME_PRODUCTION_RANGE_2_LEVEL	PREDICATE_COMPREHENSION_G	PREDICATE_COMPREHENSION_P	PREDICATE_COMPREHENSION_RANGE_2_LEVE	PREDICATE_PRODUCTION_GR
10	19	50	1	2	5	0	15	25	1	6
8	12	10	0	0	5	0	7	5	0	0
2	19	90	1	4	10	0	8	10	0	3
15	13	5	0	3	5	0	9	10	0	2
15	19	90	1	2	5	0	15	25	1	8
1	17	50	1	3	5	0	11	10	0	3

PREDICATE_PRODUCTION_PE	PREDICATE_PRODUCTION_RANGE_2_LEVEL	PCGO_DATE	PCGO_AGE	TIME_FROM_DIAGNOSIS	PCGO_GRO	PCGO_PER	PCGO_RANGE_2_LEVELS	PCGO_PART_I_GROS	PCGO_PART_I_PERC	PCGO_PART_I_RANGE_2_LEVELS	PCGO_PART_II_GROS	PCGO_PART_II_A_PERC	PCGO_PART_II_RANGE_2
5	0	04/19/2024	33	10	10	50	1	10	50	1	0	5	0
5	0	12/30/2024	21	8	1	50	1	1	50	1	0	50	1
10	0	09/25/2024	19	2	0	25	1	0	25	1	0	50	1
10	0	10/23/2024	24	15	10.5	90	1	9	90	1	1.5	90	1
10	0	07/16/2024	28	15	13	50	1	9	25	1	4	90	1
5	0	09/13/2024	26	1	4	25	1	3	25	1	1	75	1
